# Conceptualised psycho-medical footprint for health status outcomes and the potential impacts for early detection and prevention of chronic diseases in the context of 3P medicine

**DOI:** 10.1007/s13167-023-00344-2

**Published:** 2023-11-08

**Authors:** Ebenezer Afrifa-Yamoah, Eric Adua, Enoch Odame Anto, Emmanuel Peprah-Yamoah, Victor Opoku-Yamoah, Emmanuel Aboagye, Rashid Hashmi

**Affiliations:** 1 School of Science, Edith Cowan University, Joondalup, WA Australia; 2 Rural Clinical School, Medicine and Health, University of New South Wales, Kensington, NSW Australia; 3 School of Medical and Health Sciences, Edith Cowan University, Joondalup, WA Australia; 4 Department of Medical Diagnostics, College of Health Sciences, Kwame Nkrumah University of Science and Technology, Kumasi, Ghana; 5 Teva Pharmaceuticals, Salt Lake City, UT USA; 6 School of Optometry and Vision Science, University of Waterloo, Waterloo, Canada; 7 Department of Psychology, Norwegian University of Science and Technology, Trondheim, Norway

**Keywords:** Predictive preventive personalised medicine (3PM), Suboptimal Health Status Questionnaire-25 (SHSQ-25), Network analysis, Synaptic structures, LASSO model, Ghana

## Abstract

**Background:**

The Suboptimal Health Status Questionnaire-25 (SHSQ-25) is a distinctive medical psychometric diagnostic tool designed for the early detection of chronic diseases. However, the synaptic connections between the 25 symptomatic items and their relevance in supporting the monitoring of suboptimal health outcomes, which are precursors for chronic diseases, have not been thoroughly evaluated within the framework of predictive, preventive, and personalised medicine (PPPM/3PM). This baseline study explores the internal structure of the SHSQ-25 and demonstrates its discriminatory power to predict optimal and suboptimal health status (SHS) and develop photogenic representations of their distinct relationship patterns.

**Methods:**

The cross-sectional study involved healthy Ghanaian participants (*n* = 217; aged 30–80 years; ~ 61% female), who responded to the SHSQ-25. The median SHS score was used to categorise the population into optimal and SHS. Graphical LASSO model and multi-dimensional scaling configuration methods were employed to describe the network structures for the two populations.

**Results:**

We observed differences in the structural, node placement and node distance of the synaptic networks for the optimal and suboptimal populations. A statistically significant variance in connectivity levels was noted between the optimal (58 non-zero edges) and suboptimal (43 non-zero edges) networks (*p* = 0.024). Fatigue emerged as a prominently central subclinical condition within the suboptimal population, whilst the cardiovascular system domain had the greatest relevance for the optimal population. The contrast in connectivity levels and the divergent prominence of specific subclinical conditions across domain networks shed light on potential health distinctions.

**Conclusions:**

We have demonstrated the feasibility of creating dynamic visualizers of the evolutionary trends in the relationships between the domains of SHSQ-25 relative to health status outcomes. This will provide in-depth comprehension of the conceptual model to inform personalised strategies to circumvent SHS. Additionally, the findings have implications for both health care and disease prevention because at-risk individuals can be predicted and prioritised for monitoring, and targeted intervention can begin before their symptoms reach an irreversible stage.

**Supplementary information:**

The online version contains supplementary material available at 10.1007/s13167-023-00344-2.

## Introduction

### The burden of chronic diseases can be reduced in the context of predictive, preventive, and personalised medicine (3PM)

The global economic burden of chronic diseases on health services is enormous and a major public health problem. These diseases are the underlying causes of most of the mortalities and morbidities, which have been estimated to be responsible for 7 in 10 deaths each year [1, 2]. Additionally, the social cost of chronic disease care is high, as they often require ongoing monitoring and management [3]. Of particular concern are countries in sub-Saharan Africa where a significant number of adults with one or more chronic diseases currently live [2]. However, the exact prevalence of chronic diseases in these countries are unknown, because of the lack of robust systems for reliable, accurate and consistent data collection.

Evidence from the literature indicates that chronic diseases, including diabetes mellitus, heart disease, kidney disease, insulin resistance, non-alcoholic liver diseases and stroke among others, often initiate as reversible suboptimal health conditions [1]. Suboptimal health status (SHS) represents a transitional state between health and disease characterised by ambiguous health complaints in the absence of disease [4–7]. These conditions are modifiable and can be crucial for the targeted cost-effective prevention of most chronic diseases in the general population [1, 8–10]. The relevance of 3PM is that individuals at risk can be identified early for tailored management and/or treatments that would potentially prolong the onset of diseases [11–14]. Therefore, with the growing prevalence of chronic diseases, there is the need for more proactive approaches towards preventive and personalised medicine to improve the quality of life [1].

### Suboptimal health status questionnaire (SHSQ-25), an emerging tool for 3PM

One possible cost-effective approach to identifying early signs of risk is by regularly screening for persons with suboptimal health status (SHS). SHS has gained traction within the confines of 3PM, enabling the stratification of subgroups according to their risk. Yan et al. [4] designed a suboptimal health status questionnaire (SHSQ-25), which has proven to be an evidence-based and sustainable tool that is user-friendly and can provide an overall health assessment in a non-invasive manner. It comprises 25 items that determine suboptimal health by assessing five major components: fatigue, cardiovascular system, digestive system, immune system and mental health [4, 13]. The SHSQ-25 is increasingly been used as a screening tool for several chronic conditions worldwide, with Ghanaian [7], Russian [15], Chinese [13] and Korean [16] as notable examples of populations where its validity and reliability have been tested. SHS identified via SHSQ-25 has been established as a risk factor for oxidative stress [17], preeclampsia [18], type 2 diabetes mellitus [7, 19], psychological symptoms [20] and chronic stress [21]. Based on a population health survey, SHSQ-25 was identified as a viable alternative to plasma metabolites in SHS identification [22]. With the growing need for accurate prediction of diseases, SHSQ-25 has been shown to affirm the concept of 3PM, focussing on phenotypic characteristics to inform treatment and management [1].

### Working hypothesis and anticipated impact in the framework of 3PM

The synaptic connections between the 25 symptomatic items and their relevance in supporting the early monitoring and prevention of chronic diseases within the framework of 3PM remain unclear. In the pursuit of 3PM, data generated from SHSQ-25 for the general population would be deemed as ‘big data’, and therefore there is the need for robust and tailored computational methodologies that can identify the patterns of interrelatedness that exist within the five subscales to fully understand the synaptic transmission between optimal and suboptimal health outcomes. For many years, conventional statistical methods have been used to establish the relationship between health domains in the SHSQ-25 [14, 17–21]. However, data over-fitting, the curse of dimensionality and multicollinearity are only a few drawbacks that prevent effective interrogation of big data [23]. Towards this objective, computational methodologies that support data visualisation would provide an opportunity for quick, efficient and real-time monitoring of the synaptic transmissions between the health status states. Thus, we premised the present study on the hypothesis that network analysis can provide a single time point photogenic image that highlights the patterns of interactions between health status outcomes.

To be able to visualise and identify the relationships between several symptoms and their combinations associated with health status outcomes would create a comprehension for patients’ risk stratification and diagnostic paths of diseases. Network analyses have allowed researchers to visualise and identify the complex relationships between several symptoms and their combinations associated with diseases’ progression [24–27]. In network analyses, symptoms are computationally analysed, rather than relying on global scores from scales [28]. It provides data on the prognosis or outcomes for patients and utilises sum scores to demonstrate how symptoms are related to a condition. This allows for the detection of pathways by which symptoms influence each other, creating a profile of syndromes [29]. This baseline study will inform the future development of symbiotic network visualisation tools based on time-varying psychometric data from SHSQ-25 screenings. If we can construct distinctive SHSQ-25 footprint for SHS, then we have demonstrated the feasibility of creating dynamic visualisers of the evolutionary trends in the relationships between the domains of SHSQ-25 and health status outcomes, which can become a new target for 3PM with potential benefits in the healthcare systems. Thus, this approach will bring the promise of 3PM closer to translational reality from SHSQ-25 screenings to the detection and possible reversal of life-threatening conditions. More importantly, the SHS footprints would provide comprehensive evidence-based medicine that relies on sound theoretically conceptualised model that is convenient, quicker and cheaper, and would achieve personalised prediction and monitoring of suboptimal health conditions, which are precursors for chronic diseases.

## Materials and methods

### Anthropological measurements

Standard anthropological techniques were used to measure the participant’s standing height, neck, waist and hip circumference, weight and blood pressure whilst the participant was lightly clothed and shoeless. Standard equations are used to calculate body mass index (BMI), waist to hip, and waist to height ratio (WHtR).

### SHSQ-25 data collection

**Fig. 1:**
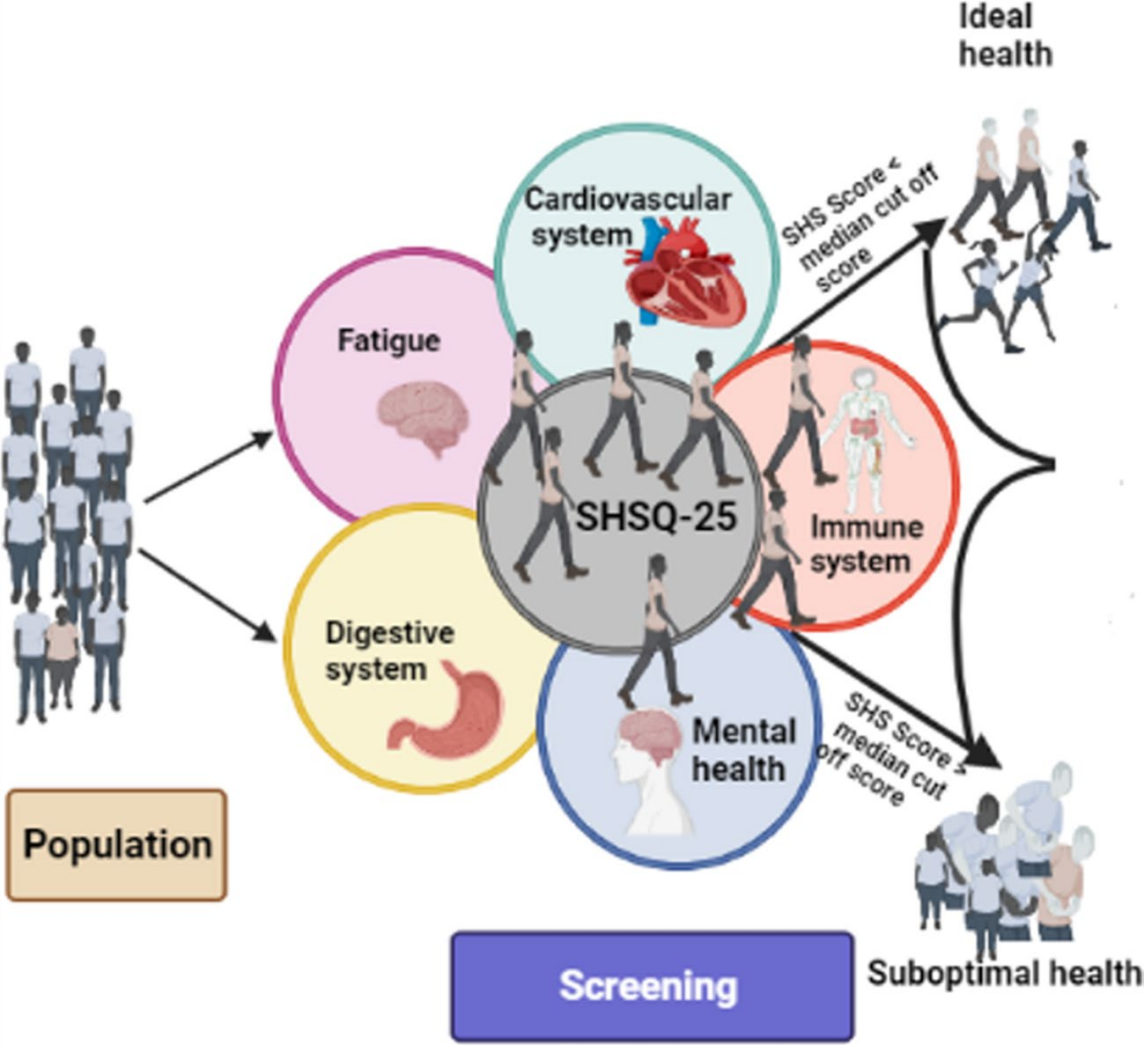
Schematic representation of the study design. The SHSQ-25 comprising of five health domains is a screening tool that can categorise individuals based on a median cut-off score. Individuals rate their health in the previous 3 months on a Likert scale, and their total SHS score is calculated. An SHS score lower and higher than the median cut-off value represents optimal or ideal and suboptimal health status, respectively

SHS is determined using a validated psychometric instrument, SHSQ-25, that comprises 25 questions which multi-dimensionally capture the health constructs of individuals from five health domains (Fig. 1): immune system (3 items), mental health (7 items), fatigue (9 items), digestive system (3 items) and cardiovascular system (3 items). Each participant was asked to rate a specific statement on a five-point Likert-type scale based on how often they suffered various specific complaints in the preceding 3 months: (0) never or almost never, (1) occasionally, (2) often, (3) very often and (4) always. Each subscale of the SHSQ-25 represents an aspect of a person’s health status which could be explored in the disease continuum. The total SHS scores were calculated for each participant by summing the ratings for the 25 items. Additionally, domain-specific SHS scores were calculated for each participant by totalling the ratings for the component subscale items. The median was used to separate low versus high total SHS scores, as has been done previously [10, 30].

### Statistical analysis—network estimation, accuracy and stability

Markov random field (MRF) is a type of stochastic process, that heavily rely on conditional probability measures, and has been widely applied in spatial statistics, and image analysis. The introduction of Gaussian Markov random fields (GMRF) has extended the scope of application to spatiotemporal statistics, structural time series analysis and graph theory (mathematical structures used to model pairwise relations) [31, 32]. A graph is made up of nodes (representing observed variables) which are connected by edges (indicating statistical relationships) [33]. The conditional probability measure in a graphical configuration is defined such that the local characteristics are only dependent on the knowledge of outcomes at neighbouring points. The restrictive version of GMRF satisfies the conditional independence assumption, such that for a given random vector ${\varvec{u}}={\left(,{u}_{2},{u}_{3}\right)}^{T}$, ${u}_{1}$ and ${u}_{2}$ are conditionally independent given ${u}_{3}$. This is true if, for a known value of ${u}_{3}$, determining ${u}_{2}$ does not inform anything about the distribution of ${u}_{1}$. Mathematically, the joint density of $\pi ({\varvec{u}})$ is given as:

$$\pi \left({\varvec{u}}\right)=\pi \left({u}_{1}|{u}_{3}\right)\pi \left({u}_{2}|{u}_{3}\right)\pi ({u}_{3})$$

An interesting property of the conditional independence assumptions of GMRF is that they impose a sparse structure (i.e. a tridiagonal form) on the precision matrix to ensure fast computation [31]. Additionally, conditional independence is applicable to both directed and undirected conditional distributions. In this study, undirected graphical models were fitted, where relationships between nodes were assumed to be symmetric. The Markov property for undirected graphical model states that for any set of nodes $T$ is independent of the rest of the graph given its neighbours:

$${U}_{T}\;{{\perp} \mskip -10.0 mu {\perp}}\;{U}_{\mathrm{non}-\mathrm{neighour}\left(T\right)}|{U}_{\mathrm{neighbour}\left(T\right)}.$$

The above relation corresponds to a factorisation of the joint distribution. The process is more complex because a symmetric neighbour of relation does not provide the opportunity to order the variables [32]. To enable factorisation of the joint distribution, the following principles from graph theory are applied: set of nodes which are neighbours known as a clique cannot be expanded and introduce potential functions ${\psi }_{c}$ that take clique configurations for a given graph $G,$ and produce non-negative numbers. We further postulate that the joint distribution approximates to a Gibbs distribution which can be expressed as:

$$p\left({U}_{1},{U}_{2}, \cdots ,{U}_{T}\right) \propto \textstyle\prod_{c\in \mathrm{cliques}\left(G\right)}{\psi }_{c}\left({U}_{i\in c}\right)$$

This indicates that the joint distribution is a product of factors, represented by the collection of cliques. We further assumed that the joint distribution of all the random variables is a multivariate Gaussian, which results in a Gaussian graphical model. Consequently, the graph can be inferred from the inverse of the covariance matrix, known as precision matrix. Due to the relatively small samples used in this study, graphical least absolute shrinkage, and selection operator (gLASSO) with tuning parameter selected by minimising the extended Bayesian Information Criterion (EBIC) was used to reliably estimate parameters represented as a weighted regularised network between observed variables, and further evaluated the robustness and accuracy of the network structure and network parameters via measures from graph theory [32–35].

The accuracy of the connections in the network structures was evaluated by assessing the properties of the edges’ weights via bootstrapping [33]. The 95% confidence intervals of the edges’ weights were constructed to reveal patterns of overlaps or otherwise. We further assessed the patterns of node placements via multi-dimensional scaling (MDS)-based algorithms [36]. The network stability was evaluated to ascertain their robustness to sampling variations, using a drop and re-estimate scheme called sub-setting bootstrapping [28, 37]. We conducted the routine implemented in *bootnet* package [33], using nonparametric bootstrapping based on 500 bootstrap samples. We further examined the stability of the order of centrality indices, by looking at the correlations between centrality indices of network resulting from dropping substantial number of cases and the centrality indices of the original network. For central indices to be considered stable, the centrality stability coefficient must be at least 0.25 and preferably above 0.50. We further examined the node predictability to understand the shared variance properties of the networks.

For network estimation, we used the *estimateNetwork* function in the ‘bootnet (v 1.5)’ package [33], using the *EBICglasso* function from ‘qgraph (v 1.9.3)’ package [38] in the R software [39]. The accuracy of estimates has been assessed using the bootnet function. Networks have been visualised using the ‘qgraph’ package [38].

## Results

### Participants’ characteristics

The study involved 217 participants aged between 30 and 80 years, with an average age of 55.09 (± 9.27), and females constituting 61.2%. Table 1 presents the sample distributions and baseline measured clinical features between the optimal and suboptimal populations. In general, the suboptimal population was comparatively older and had significantly higher scores for both systolic and diastolic blood pressure (*p* < 0.05), with small to medium effect sizes. No statistically significant differences were observed between the optimal and suboptimal populations for BMI, height, weight, waist and hip circumference and WHtR (*p* > 0.05).

**Table 1:** Summary statistics on demographic and anthropometric data of study participants

Variables	Optimal (*n* = 106)	Suboptimal (*n* = 111)	Remarks (*p*-value and effect size)
Gender			
Female	59 (55.66%)	74 (66.67%)	
Male	47 (44.34%)	37 (33.33%)	
Marital status			
Single	3 (2.83%)	13 (11.71%)	
Married	86 (81.13%)	62 (55.86%)	
Divorced/Separated	8 (7.55%)	12 (10.81%)	
Widowed	11 (10.38%)	23 (20.72%)	
Education			
No formal	4 (3.77%)	12 (10.81%)	
Primary	9 (8.49%)	20 (18.02%)	
High school	74 (69.81%)	65 (58.56%)	
Tertiary	19 (17.92%)	10 (9.01%)	
**Age**	**53.09 ± 7.57**	**57.02 ± 10.43**	***p* = *0.002; d* = *0.43 (medium*)**
31–50	43 (40.57)	34 (31.20%)	
51–70	61 (57.55)	63 (57.8%)	
71 +	2 (1.89%)	14 (11.00%)	
BMI (kg/m^2^)	25.67 ± 4.78	25.56 ± 5.04	*p* = 0.869; *d* = 0.02 (small)
Underweight	4 (3.77%)	9 (8.11%)	
Normal	43 (40.57%)	46 (41.44%)	
Overweight	41 (38.68%)	33 (29.73%)	
Obese	18 (16.98%)	22 (19.82%)	
**Systolic BP (mmHg)**	**142.06 (22.86)**	**149.70 (25.31)**	***p* = *0.021; d* = *0.32 (small)***
Normal	32 (30.19%)	24 (21.62%)	
High SBP	74 (69.81%)	87 (78.38%)	
**Diastolic BP (mmHg)**	**82.52 (12.99)**	**86.58 (15.54)**	***p* = *0.038; d* = *0.28 (small)***
Normal	59 (55.66%)	54 (48.65%)	
High DBP	47 (44.34%)	57 (51.35%)	
Height	1.64 ± 0.07	1.61 ± 0.07	*p* = 0.837; *d* = 0.03 (small)
Weight	68.70 ± 12.78	66.49 ± 14.40	*p* = 0.234; *d* = 0.16 (small)
Waist circumference	89.72 ± 12.57	90.86 ± 12.95	*p* = 0.512; *d* = 0.09 (small)
Hip circumference	101.22 ± 10.82	101.20 ± 10.91	*p* = 0.989; *d* = 0.002 (small)
WHtR	0.55 ± 0.08	0.56 ± 0.08	*p* = 0.358; *d* = 0.13 (small)

NB: Bold text indicates significant difference at 5% level of significance. Effect size measures the practical significance (meaningfulness) of difference between groups

### SHSQ-25 response distributions for optimal and suboptimal populations

Regarding participants’ responses to the SHSQ-25 questionnaire items, SHS population observed higher mean responses compared to the optimal population across all subscales, indicating that the suboptimal population often reported of occasionally, and/or often, and/or very often, and/or always experienced the specified symptoms (Table 2). The nominal differences in the responses for the items under the domain ‘fatigue’ were comparatively prominent between optimal and suboptimal populations. The suboptimal population overwhelmingly reported that they often or very often or always felt exhausted without increasing their physical activities, expressed headaches, expressed pains in shoulder, neck and back and experienced muscle stiffness.

**Table 2:** Descriptive analyses of the distribution of item responses in SHSQ-25 among the optimal and suboptimal study populations

Domain	ID: Question items	Optimal (*n* = 106)	Suboptimal (*n* = 111)
		Mean (SD)	Mean (SD)
Cardiovascular system (CS)	CS1: How often did you feel out of breath whilst resting?	0.11 (0.37)	0.94 (0.85)
	CS2: How often did you suffer from chest congestion?	0.10 (0.36)	1.13 (1.10)
	CS3: How often were you bothered by heart palpitations?	0.37 (0.81)	1.59 (1.23)
Digestive system (DS)	DS1: How often was your appetite poor?	0.14 (0.52)	0.75 (0.97)
	DS2: How often did you suffer from heartburn?	0.28 (0.77)	1.23 (1.15)
	DS3: How often did you suffer from nausea?	0.23 (0.63)	0.96 (1.04)
Fatigue (FT)	FT1: How often were you exhausted without greatly increasing your physical activity	0.64 (1.01)	2.20 (1.07)
	FT2: How often did you have fatigue which could not be substantially alleviated by rest?	0.34 (0.59)	1.32 (0.95)
	FT3: How often were you lethargic in your daily life?	0.34 (0.65)	1.26 (0.91)
	FT4: How often did you suffer from headaches?	0.75 (0.96)	2.14 (1.28)
	FT5: How often did you suffer from dizziness?	0.28 (0.58)	1.52 (1.24)
	FT6: How often did your eyes ache or feel tired?	0.37 (0.72)	1.58 (1.17)
	FT7: How often did your muscles or joints feel stiff?	1.02 (1.15)	2.10 (1.03)
	FT8: How often did you have pain in your shoulders /neck /back?	0.79 (1.03)	2.14 (0.90)
	FT9: How often did you have a heavy feeling in your legs when walking?	0.42 (0.77)	1.60 (1.22)
Immune system (IS)	IS1: How often did you have difficulty tolerating hot and cold temperatures?	0.15 (0.39)	1.15 (0.96)
	IS2: How often did you catch a cold?	0.51 (0.81)	1.43 (1.10)
	IS3: How often did you suffer from a sore throat?	0.22 (0.57)	1.17 (1.17)
Mental health (MH)	MH1: How often did you have difficulty falling asleep?	0.39 (0.85)	0.98 (1.14)
	MH2: How often were you troubled by waking up during the night?	0.29 (0.65)	1.19 (1.11)
	MH3: How often did you have trouble with your short-term memory?	1.15 (1.21)	2.07 (1.25)
	MH4: How often did you did you have difficulty responding to situations quickly or making decisions?	0.42 (0.63)	1.14 (0.89)
	MH5: How often did you have difficulty concentrating?	0.43 (0.76)	1.17 (0.99)
	MH6: How often were you distracted for no reason?	0.33 (0.69)	1.06 (1.02)
	MH7: How often did you feel nervous or jittery?	0.34 (0.80)	1.37 (1.30)

**Fig. 2:**
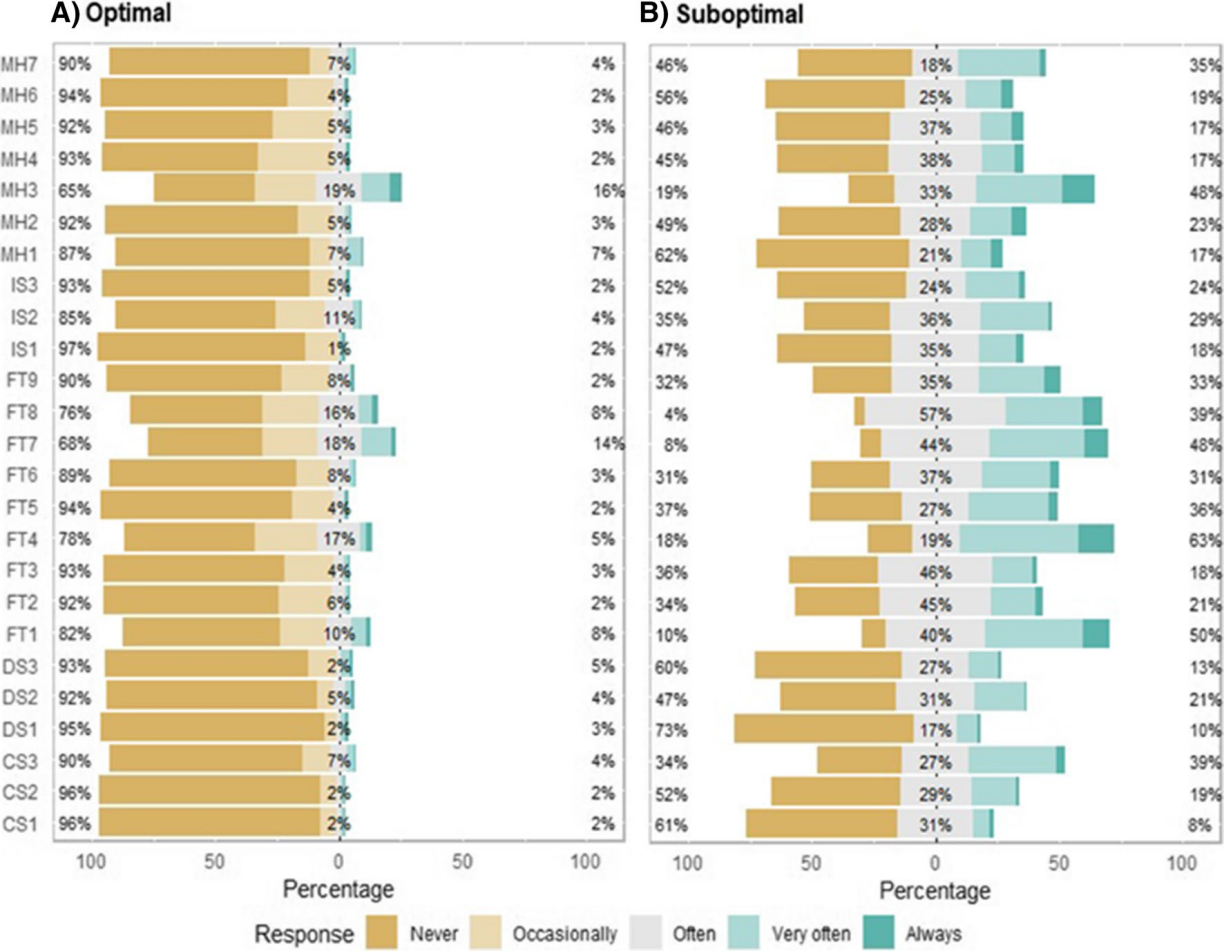
Item by item response distribution for the optimal and suboptimal population cohorts

The expression of symptoms associated with immune system, cardiovascular systems and digestive system were minimally reported among the optimal population compared to the suboptimal population (Fig. 2; Figure S1 of supplementary material). It can be observed from the optimal population response profile that some of the individuals in the cohort did not express or/and experience any of the symptoms across the five domains of SHSQ-25 (Fig. 2a). However, almost all individuals in the suboptimal population expressed or/and experienced the symptoms across all the five domains of the SHSQ-25 in varying degrees (Fig. 2b).

### SHSQ-25 domain network for optimal and suboptimal populations

**Fig. 3:**
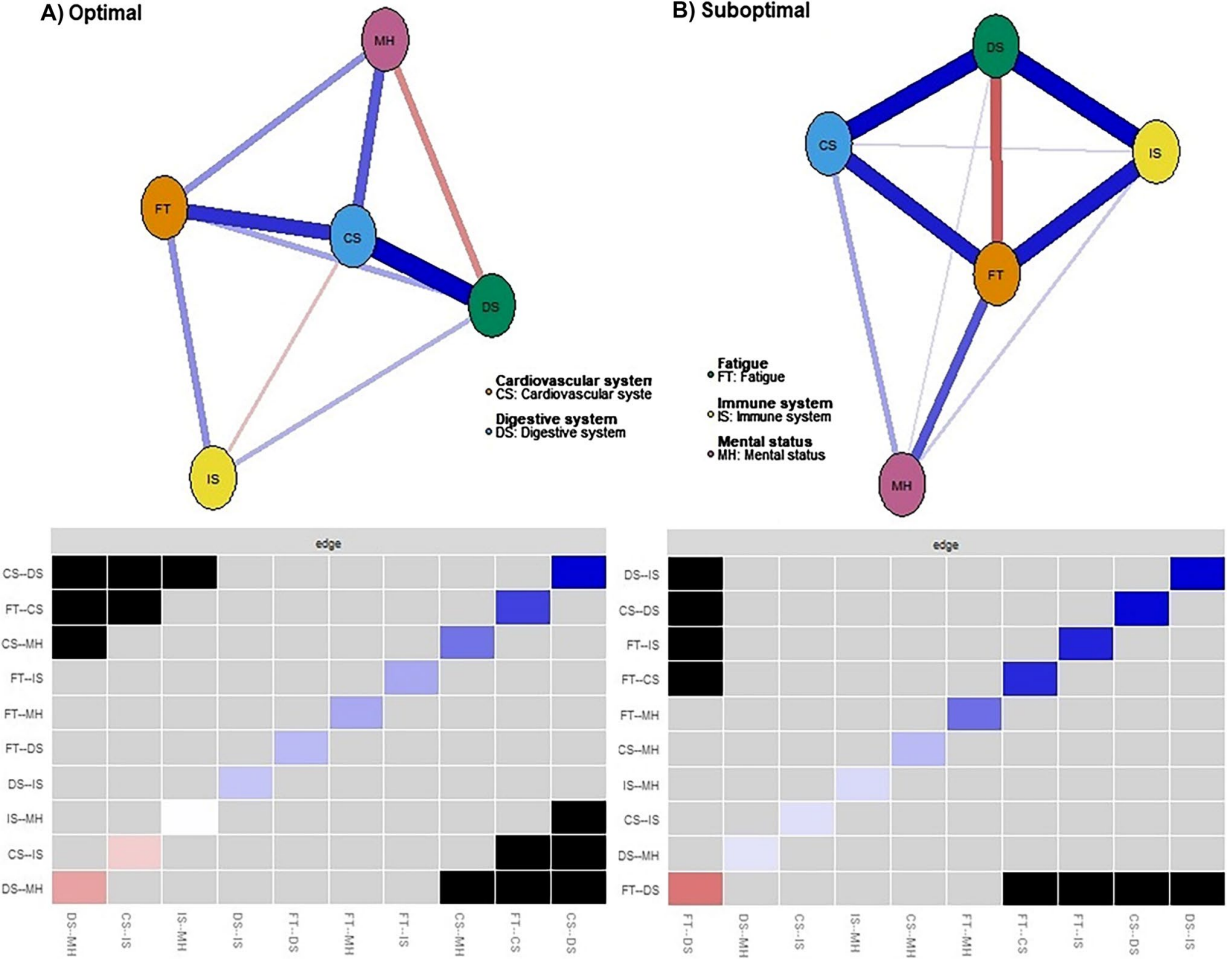
Five-domain networks of SHSQ-25 for **A)** optimal population and **B)** suboptimal population with bootstrapped difference tests (*α* = 0.05) between edge weights in the estimated networks. Gray boxes indicate edges that do not differ significantly from one another, and black boxes represent edges that do differ significantly from one another. Blueish-shaded boxes on the main diagonal indicate positive connections between nodes; brownish-shaded boxes indicate negative connection, the darker the blue or brown the stronger the positive or negative correlation. The white shaded box indicates no connection between nodes

The five-domain networks of the SHSQ-25 for the optimal and suboptimal populations are presented in Fig. 3. The 25 items in SHSQ-25 are represented as nodes, and the relationships among them are expressed as edges to reveal the temporal patterns of disease development. The edges in the networks represent the conditional partial correlations obtained via the extended BIC criterium graphical lasso (EBICglasso) method with tuning parameter set to 0.5 [40] and are presented in the Fruchterman-Reingold algorithm layout [41]. Both networks are composed of positive and negative connections between domains. *Cardiovascular system (CS)* was the most centrally placed domain for the optimal population network, whilst *fatigue (FT)* was for the suboptimal population network. In the optimal population network, *cardiovascular system* shared strong positive connections with *fatigue (FT), digestive system (DS)* and *mental health (MH)* and a weak negative connection with *immune system (IS)*. All other connections were weakly positive except for the moderate negative connection between *mental health* and *digestive system* and a statistically independent relation between *immune system* and *mental health*. The network structure for the suboptimal population network is more dense with relatively strong connections between domains. For example, strong positive connections were observed between the following pairs: *fatigue*–*cardiovascular system*, *fatigue*–*immune system, digestive system*–*immune system,* and *cardiovascular system*–*digestive system*. A strong negative connection between *fatigue* and *digestive system* was observed. Unlike the optimal population network, shared connections were observed between all paired domains in the suboptimal population network.

### SHSQ-25 domain networks—node predictability and stability

The correlations among the centrality indices were relatively stronger in the suboptimal population network compared to the optimal population network (0.87 vs 0.76 for betweenness and closeness, 0.83 vs 0.71 for strength and closeness and 0.84 vs 0.68 for strength and betweenness). The node predictability scores for the optimal population domain network were FT = 0.61; CS = 0.78; DS = 0.67; IS = 0.56; and MH = 0.48 with an average of 0.62 (CS > DS > FT > MH > IS. On the other hand, the node predictability scores in the suboptimal network were FT = 0.81; CS = 0.68; DS = 0.74; IS = 0.64; and MH = 0.39 with an average of 0.65 (FT > DS > CS > IS > MH). These statistics indicate that 62% and 65% of variances in the nodes can be explained by neigbouring nodes in the optimal and suboptimal networks, respectively.

The global variance test within the *Network Comparison Test* procedure indicated there were some differences in the overall level of connectivity between the optimal and suboptimal population networks (*p* = 0.007). The weighted adjacency matrices showed a large correlation (*r* = 0.760), indicationg some level of similarity in the overall structure of the networks. This reveals that the two networks differed in the strength of connectivity, with some edges showing similar patterns. For instance, the edge strength for *fatigue*–*cardiovascular system* and *cardiovascular system*–*digestive system* are identical in both networks.

### SHSQ-25 item network, node predictability and stability for optimal and suboptimal populations

**Fig. 4:**
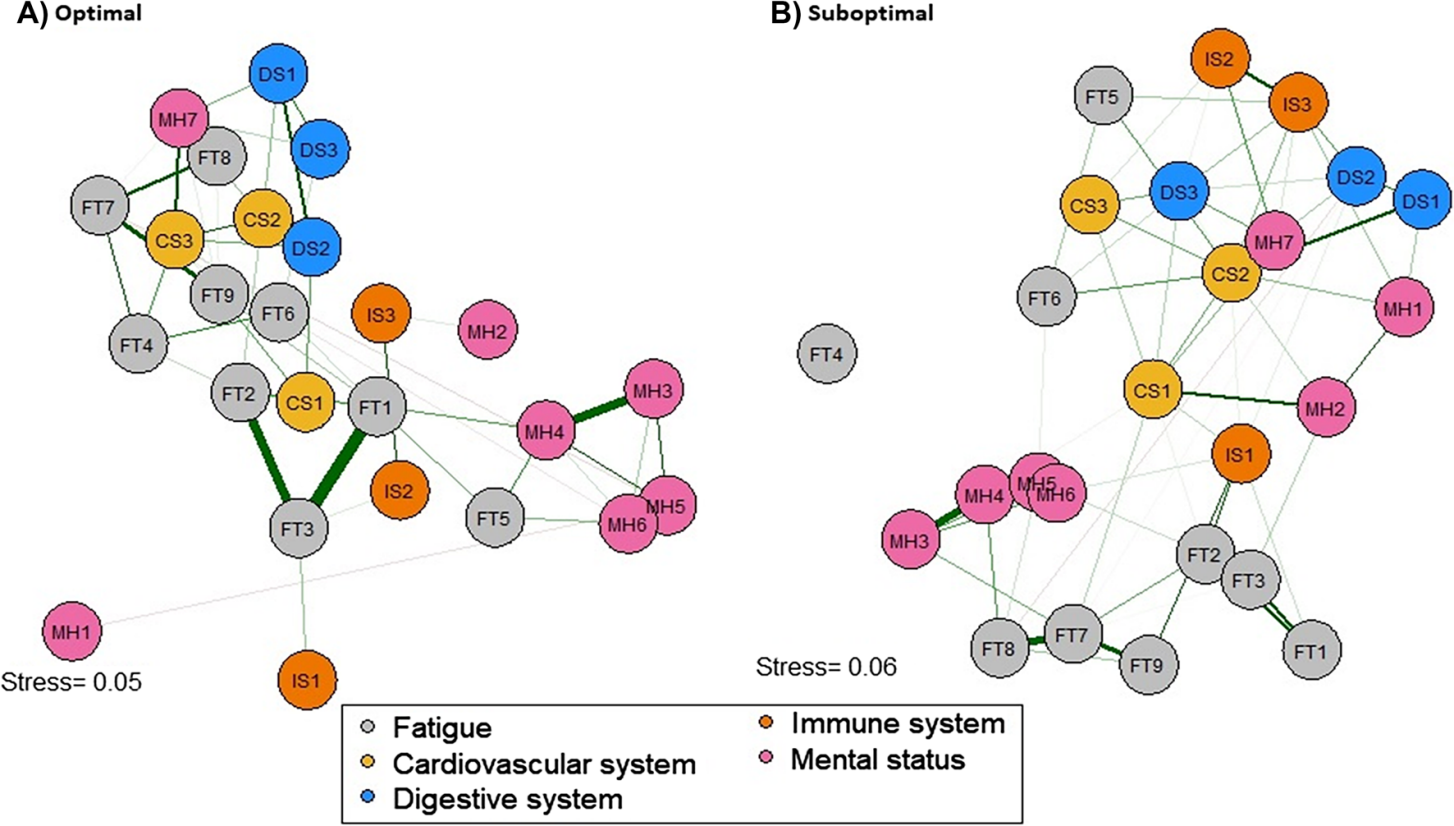
Graphical LASSO network for **A)** optimal and **B)** suboptimal population cohorts, plotted with ordinal MDS configuration based on zero-order correlations for the 25 items of the SHSQ-25 questionnaire plotted as nodes. Green edges (i.e. connections) represent positive associations and red edges represent negative association. The thicker the connection, the stronger the association between nodes. Colour codes represent the 5 domains: *FT *Fatigue, *MH *Mental health, *IS* Immune system, *DS* Digestive system, *CS* Cardiovascular system

In a more detailed analysis of the SHSQ-25 items, the ordinal (see Figs. S2 and S3 for Shepard diagrams) MDS-based LASSO algorithm revealed structural, node placement and node distance differences between the networks for the optimal and suboptimal populations (Fig. 4). A central assumption for the item network models is the assumption of sparsity. The optimal population network had 58 non-zero edges identified out of possible 300 (network density of 0.193 with mean edge weight of 0.043), whilst 43 non-zero edges were identified in the network for suboptimal population (network density of 0.143, with mean edge weight of 0.024). Distinct structures and node placements were observed between the networks, with different but relatively low-stress values. Node predictability ranged from 0.24 to 0.97, with an average of 0.64 for the optimal population network, whilst that for suboptimal ranged from 0.08 to 0.98, with an average of 0.67. These indicate that on average 64% and 67% of variances in the nodes, respectively, were explained by neigbouring nodes for the optimal and suboptimal networks. ‘FT3’ had the highest node predictabilty (0.97) in the optimal population network, followed by ‘MH3’(0.96) and ‘FT2’(0.94). In the suboptimal network, ‘MH4’ was the highest predictable node (0.97), followed by ‘MH5’ (0.97) and ‘FT7’ (0.95).

**Fig. 5:**
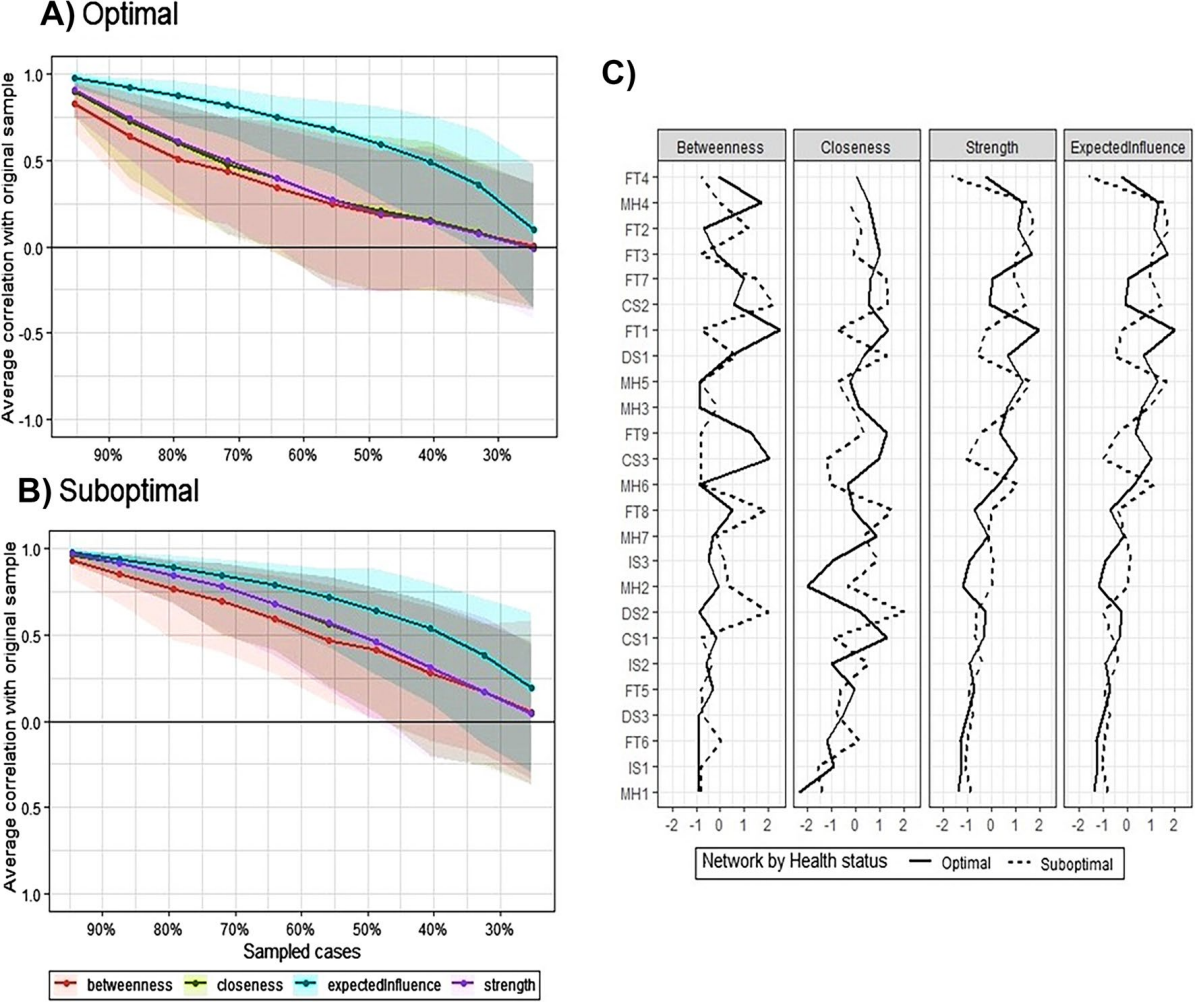
Average correlations between centrality indices of **A)** optimal and **B)** suboptimal population networks sampled with samples dropped and the original sample. Bold lines indicate the means of the various centrality indices, and the areas indicate 95% confidence range. **C)** Centrality indices for the 25 items in the networks presented in Fig. 4, which are shown as standardised *z*-scores

Overall, the clique (or cluster) configurations in both networks reflect the conceptual framework of the SHSQ-25 to a larger extent. Notable items which deviated from their cliques (or clusters) in both networks include ‘IS1’, ‘MH1’ and ‘MH7’. ‘MH7’ in both networks was identified to be closely related to the ‘*cardiovascular system*’ domain than ‘*mental health’*. Item ‘IS1’ was more related to the ‘*Fatigue*’ domain for both populations. ‘FT4’ in the suboptimal network was conditionally independent of all other nodes. In both networks, within-clique connections were positive, with varying strengths (mostly weak). Whilst there was a weak negative connection between ‘DS2’ and ‘FT8’ in the suboptimal population netowrk, the two nodes were conditionally independent in the optimal population network. The domains ‘*mental health*’ and ‘*fatique*’ had sub-cliques in both networks but were more apparent in the suboptimal population network. For instance in the suboptimal network, items ‘MH3’, ‘MH4’, ‘MH5’ and ‘MH6’ were closely knit together, whilst ‘MH1’, MH2’ and ‘MH7’ form an independent clique, for the ‘*mental health*’ domain. In the optimal population network, items ‘MH3’, ‘MH4’, ‘MH5’ and ‘MH6’ formed a clique, but ‘MH1’, MH2’ and ‘MH7’ were independent of each other. The sub-domain clique configurations for ‘*fatique*’ was slightly different between the optimal and suboptimal networks. Whilst ‘FT1’, FT2’ and ‘FT3’ were closely knit in both networks, ‘FT4’, ‘FT6’, ‘FT7’, ‘FT8’ and ‘FT9’ formed a sub-clique in the optimal population network, with only FT7’, ‘FT8’ and ‘FT9’ forming the sub-clique in the suboptimal population network. Similar to the domain networks, the correlations among the centrality indices were relatively stronger in the suboptimal population network compared to the optimal population network (0.82 vs 0.69 for betweenness and closeness, 0.75 vs 0.64 for strength and closeness and 0.73 vs 0.59 for strength and betweenness). ‘FT1’ and ‘FT3’ were the top two nodes with the highest strength in the optimal population network, whilst ‘FT2’ and ‘MH5’ were for the suboptimal population network (see Fig. 5C).

The global variance test indicated there were some differences in the overall level of connectivity between the optimal and suboptimal population networks (*p* = 0.024). The weighted adjacency matrices showed a moderate correlation (*r* = 0.630), indicationg some level of similarity in the clique configuration of the networks. On accuracy and stability, the 95% confidence intervals of the edge weights were generally narrow, indicating high level of stability in the results (see Fig. S4 of supp material). Additionally, the strength centrality estimates were moderately stable, with centrality stability coefficients of 0.51 and 0.54 for the optimal and suboptimal population networks, respectively. These estimates indicate that 51% and 54% of respective data could be dropped to retain a correlation of 0.7 with the original dataset at a 95% certainty level (see Fig. 5A and B).

## Discussion

### Summary of research findings

Accurate prediction of health status outcomes is fundamental to the concept of 3PM. SHSQ-25 provides an avenue for making early judgement on health status outcomes to inform tailored interventions to promote the wellbeing of the general population. The study demonstrates the practical application of the SHSQ-25 for early identification of suboptimal health outcomes, which would provide an opportunity for personalised management, to avert the development of chronic conditions. We have explored the internal structure of SHSQ-25, highlighting the diverse relationship patterns that reveal the footprints for optimal and SHS outcomes and established the discriminative capabilities to categorise these two groups. We examined the 5-domain and 25-item network structures of the SHSQ-25 to map the interrelationships and centrality of the health status outcomes. Whilst prior studies have established the face, content and construct validity of the SHSQ-25 across Chinese and Ghanaian populations [6, 42], the current study has been extended to explore the patterns of covariation on the premise that items can mutually influence one another due to their conditional dependence on the latent variables [33]. The analytical approach used provided the opportunity to thoroughly investigate both item-level and domain-level perspectives of the internal structures and centrality placements for optimal and suboptimal population to provide clinical insights into appropriate intervention approaches. For example, we observed differences in the cluster (or clique) formations, nature and strength of associations and exact placement of nodes in the network structures for optimal and suboptimal populations (Fig. 4). The clique formations and strongest edges (which were mostly positively connected) in the items networks largely confirmed the theoretical subclinical conditions (i.e. immune system, mental health, fatigue, digestive system and cardiovascular system) hypothesised as the pillars for measuring health status outcomes.

### Construction of synaptic networks of health status outcomes within the framework of 3PM

Applying network analysis, the present study has established the interrelationship and centrality of the items in the five health domains of the SHSQ-25. The results of the study showed that the cardiovascular system domain had the greatest relevance for optimal health, evidenced by the many connections or edges. This domain measured an individual’s heart palpitations, heart burns, nausea, difficulty tolerating cold and hot temperatures and shortness of breath. When compared to the optimal health individuals (low SHS), those with suboptimal health had higher scores for heart palpitations. Defined as abnormally irregular heartbeat, they are characterised by a pounding sensation of the chest, rapid fluttering or flip flopping in the chest and a perception of skipped beat. Heart palpitation can indicate the underlying cause of a life-threatening condition. Indeed, it has been linked to mental health conditions such as depression, panic attacks and generalised anxiety disorders among others. Whilst heart palpitations can be caused by factors such as nicotine use, emotional stress, exercise, fever and alcohol, heart palpitation can also be due to underlying medical conditions, such as high blood pressure and coronary heart disease [43, 44].

The domain network structures (Fig. 3) and taxonomic profiles (Figure S1 of supplementary material) highlighted fatigue as being the most centrally placed domain for the suboptimal population, suggesting it plays an important role in the network and its activation has a greater influence on other nodes. The connection between fatigue and chronic diseases is well established in the literature [45–49]. For example, patients with functional chronic gastrointestinal (GI) disorders were found to be more fatigued than the control group, as well as a group that were diagnosed to have organic GI disease [45]. A population-based Lifelines Cohort Study of 78,363 subjects showed that a higher proportion of participants with one or more chronic conditions were severely and chronically fatigued [47]. Among patients with chronic inflammatory and autoimmune diseases, there is an upward trajectory of fatigue complaints. This was highlighted in the study by Skjellerudsveen et al. [49], who reported that the 41 to 50% of patients with newly diagnosed celiac disease had the presence of clinically relevant fatigue. However, health care professionals have long ignored the complaints of fatigue and overly concentrated on often expensive ‘hard, objective disease endpoints’ (such as clinical biomarkers) because of the subjectivity of fatigue and the lack of therapeutic treatment [46]. To this end, instruments that effectively measure fatigue such as SHSQ-25 would be instrumental in the space of proactive healthcare that personalises the prevention, stratification, diagnosis and treatment for specific patients. Understanding fatigue as a health construct has greater potential of helping clinicians understand complex chronic syndromes posing a greater burden to health care delivery [48].

### Conceptual model of SHSQ-25—a paradigm shift from reactive to 3PM and moving beyond the state of the art

SHSQ-25 was developed from themes related to a broad range of perceived health complaints among 3000 seemingly healthy persons via focus group discussions [6]. The concept of suboptimal health has been viewed as a new dimension for translational medicine, since by characterising consistently functional and metabolic efficiency parameters could support the diagnosis of chronic diseases such as cancer, diabetes, and hypertension [1, 5]. Apparently, the five identified domains holistically evaluate health and wellbeing via 25 items strategically defined to probe the functional and metabolic efficiency of the individual. These items are deemed to be interrelated, and the extreme range of responses highlights the likelihood of optimal and suboptimal health. Overall, we found positive associations at domain-level and item-level evaluation across the optimal and suboptimal population cohorts, with some few relatively weak negative associations. For instance, at the domain-level association, mental health and digestive system, and cardiovascular system and immune system were negatively correlated for optimal population whilst similar trend was found between the digestive system and fatigue in the suboptimal population (Figs. 3 and 4).

The synergetic strength of the five domains of SHS has been established in their patterns of associations with several chronic conditions including cardiovascular diseases, type II diabetes mellitus, preeclampsia and psychological symptoms in studies conducted in different geographical contexts [7, 15, 50]. For example, Kupaev et al. [15] noted in a Russian population that endothelial dysfunction was negatively associated with three of the domains of SHS, namely, fatigue, mental health and cardiovascular system, highlighting the positive relationships between domains. Similar findings were reported in Hou et al. [50] for a Chinese Han population, where they established varying positive relationships between a range of psychological symptoms and the five domains of SHS. Using the Symptom checklist-90, they assessed participants’ psychological symptoms including somatisation, obsessive compulsive, interpersonal sensitivity, depression, anxiety, paranoid ideation and psychoticism). In a different Ghanaian population, Adua, Roberts and Wang [7] and Anto et al. [18], respectively, found associations between the SHS and type II diabetes mellitus and pregnancy disorder condition preeclampsia. Notably, Anto et al., [18] observed that the incidence of preeclampsia increased with increasing SHS-specific domain score for fatigue, cardiovascular complaints, digestive system disorder, immune health disorder and mental health complaints.

### Predictive personalised medicine and targeted prevention approach

The SHSQ-25 has thus far been shown to predictively identify the early signs of risk in a general population. This would be beneficial, as screening would target a specific population, whilst reducing screening for lower-risk individuals. For example, given the genetic diversity within a population, patients with metabolic conditions may have the same clinical profile (e.g. body mass index, glycated haemoglobin, age, plasma lipid status) but will respond differently to a treatment. Thus, healthcare professionals must examine the genetic makeup of each individual and develop therapies that are specific to that individual. Moreover, people who complete the SHSQ-25 will know their risk, which in turn would empower them to modify their lives in a manner that would reduce their risk or protect them from transitioning into a disease [14, 30].

The path to developing a chronic disease can be long, even up to 30 years [5, 14]. Thus, from the optics of the 3PM point of view, the current approach of treating chronic conditions after symptom onset is a delayed response. SHS screenings at healthcare facilities observed over time would provide rich data gathered economically [51]. The proposed method of analysis provides opportunity to provide holistic care, which will be cost-effective in determining individualised health status, and initiates an intervention before symptoms worsen. The foundation of holistic care is that the manifestation, severity and long-term effects of a particular chronic condition can be prevented [14, 30]. To combat the delayed intervention, untargeted medication, overdosed and poisoned patients and poor therapy, primary care practitioners must be able to recognise and manage SHS. This must go beyond the usual recognition of physical deterioration, but also practitioners must pay attention to the psychological state of the individual. The SHSQ-25 offers the opportunity to recognise the reversible damage in an individual and serves as a catalyst to establish appropriate interventions for risk reduction [4–7].

## Limitations

The main drawback of network theory is that it is not always obvious how powerful the various interactions and influences are both inside and between different networks. As a result, even whilst significant interactions between the different SHS domains have been observed, it remains unknown how practical these interactions are. Nonetheless, network analysis could expand on the variables within the SHS health domains, providing insights into how multiple variables interact to cause a disease. Expanding on our findings will require further research that uses a wider range of characteristics related to chronic illnesses and more diverse samples from different populations. It must be noted that this was a cross-sectional study and did not allow us to measure accurate causality between the subclinical symptoms and chronic diseases. Additionally, all data were collected by self-reported questionnaires, which may have some propensity towards information bias.

## Outlook and expert recommendations in the framework of 3PM

Applying network analysis to SHSQ-25 screening data has allowed for the detailed exploration of the relationship patterns among the five health domains into early detection. Effectively, the SHSQ-25 instrument has been verified for being robust, consistent and reliable. This baseline study has extended the narrative and provided extensive investigation of the cross-sectional footprints of the five SHS dimensions among a suboptimal population cohort, providing many insights into the feasibility and opportunities of longitudinal study designs. Nonetheless, in the pursuit of 3PM, the administration of SHSQ-25 must be made in conjunction with person-specific information, as well as objective biomarkers including genes (e.g. inherited genetic mutations), mRNAs, proteins, glycans and lipids. When these biomarkers are combined with subclinical phenotypes, a holistic SHS diagnosis can be established for use in 3PM of chronic diseases [52, 53]. The availability of next-generation sequencing technologies and state of the art techniques including tandem mass spectrometry, ultraperformance liquid chromatography and capillary gel electrophoresis have made the quantification and detection of metabolites seamless [54–56]. When this information is obtained, we could employ the use of network analysis to determine the protein–protein interactions, metabolic networks, gene regulatory networks and signalling information in biological networks in an individual. Triangulating data generated from these methodologies with network analysis would be a catalyst to drive the development of new treatments. Going forward, machine learning (ML) approaches could also be leveraged to drive the agenda of 3PM. Better health planning, disease forecasting and disease risk characterisation can all be possible by using it to translate such data. ML techniques can evaluate and query the data in a way that was previously not possible using traditional statistical techniques, to promote accurate prediction of diseases to allow the identification of vulnerable people and target them for treatment.

It is important to highlight that the far-reaching impact of the SHSQ-25 in mitigating the challenges arising from language barriers is now evident, with the SHSQ-25 successfully translated into Korean, Chinese and Russian languages [4, 15, 16]. Effective communication is critical to drive the concept of the 3PM. Central to effective communication is a language, which allows interacting individuals to understand each other. When a language barrier exists, an interpreter or a translator is recommended to address the issues with communication. However, this comes with a significant challenge, as hiring an interpreter comes with a cost and some information may be lost in translation. Besides, there is a tendency to breach patient confidentiality, making patients keep information to themselves rather than sharing it with the health professional. This potentially leads to misdiagnosis and unnecessary medications. All these can negatively impact healthcare delivery and adversely affect the relationship between the healthcare professional and the patient. The emergence of the SHSQ25, already translated into a local language including Russian, Chinese and Korean, can help mitigate these challenges and stimulate the 3PM concept. When individuals understand the questions being asked, they can provide the right information to get the appropriate treatment or advice.

## Conclusions

The fact that 3PM is extremely adaptable to advanced techniques and statistical methods makes it an excellent concept to promote health and improve the quality of life of people. With the growing variety and quantum of medical data, the field is now heavily relying on the expertise of quantitative analysts to make meaning out of the ‘big data’. Data visualisation in medical research has become useful in disease diagnosis, as it provides opportunity for quick, efficient and often real-time monitoring of the aetiological processes of diseases. To visualise and identify the relationships between several symptoms and their combinations associated with diseases create a comprehension for patients’ risk stratification and diagnostic paths of diseases. The ability to visualise and identify the relationships between several symptoms and their combinatory associations with diseases would create an in-depth comprehension for patients’ risk stratification and diagnostic pathways for diseases, and these align well within the framework of 3PM.

Based on analysing a cross-sectional SHSQ-25 screening data, the taxonomic profile and domain network structure highlighted fatigue as being the most prevalent and central subclinical condition to the suboptimal population, whereas the cardiovascular system domain had the greatest relevance for optimal health population. The successful applications of SHSQ-25, along with complex network analysis of patient or individual profiles, are central to health promotion and lead to cost-effective targeted prevention. The SHSQ-25 is a simple tool that can be self-administered prior to or during a consultation with a medical practitioner [12]. The outcome of this study falls within the framework of 3PM, in that we have proven the effectiveness of the SHSQ-25 to identify people with symptoms that are yet to meet the diagnostic criteria of a chronic disease. Particularly within the scope of primary and secondary prevention, the SHSQ-25 has been proven to be a useful resource that maximises health outcomes by identifying at-risk individuals or people at the subclinical stage of a chronic condition. The baseline study has the potential to inform future longitudinal study on SHSQ-25 as a ‘pseudo marker’ and a vehicle to drive the transition from reactive medical services to 3PM.

### Supplementary Information

Below is the link to the electronic supplementary material.

Supplementary file1 (DOCX 1341 KB)

Supplementary file2 (DOCX 18 KB)

## Data Availability

The statistical codes and dataset analysed during the current study are available from the corresponding authors on reasonable request.

## Abbreviations

*BMI*: Body mass index
*CS*: Cardiovascular system
*DS*: Digestive system
*EBIC*: Extended Bayesian Information Criterion
*EBICglasso*: Extended BIC criterium graphical least absolute shrinkage, and selection operator
*FT*: Fatigue
*GI*: Gastrointestinal
*gLASSO*: Graphical least absolute shrinkage, and selection operator
*GMRF*: Gaussian Markov random field
*IS*: Immune system
*MDS*: Multi-dimensional scaling
*MH*: Mental health
*ML*: Machine learning
*mmHg*: Millimetres of mercury
*MRF*: Markov random field
*PPPM/3PM*: Predictive, preventive, and personalised medicine
*rRNA*: Messenger Ribonucleic acid
*SHS*: Suboptimal Health Status
*SHSQ-25*: Suboptimal Health Status Questionnaire-25
*WHtR*: Waist to height ratio
