# Supplementary material for: Conceptualised psycho-medical footprint for health status outcomes and the potential impacts for early detection and prevention of chronic diseases in the context of 3P medicine
Source: EPMA J. 2023 Nov 8;14(4):585–99. doi: 10.1007/s13167-023-00344-2 (PMC10713508; doi:10.1007/s13167-023-00344-2)
Supplement: Supplementary file 1 — Supplementary file1 (DOCX 1341 KB) [file 13167_2023_344_MOESM1_ESM.docx]

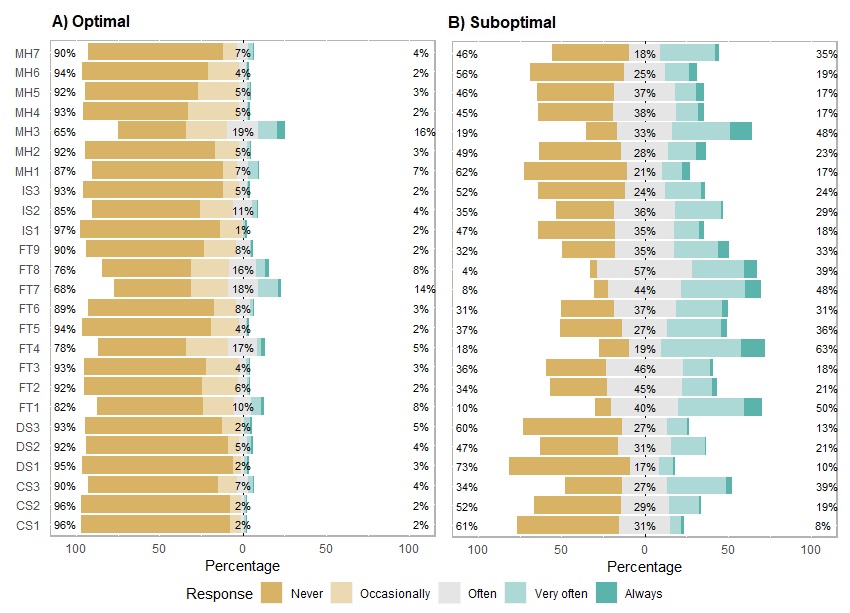


Figure S1: Item by item response distribution for the optimal and suboptimal population cohorts


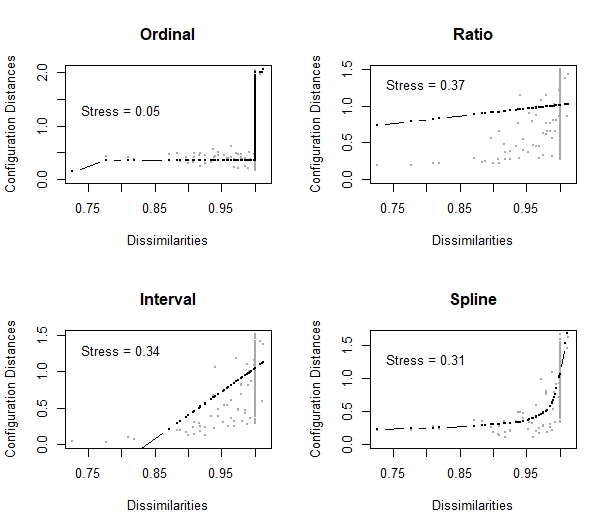


Figure S2: Shepard diagrams associated with the optimal population cohort. These are metric information in the proximities to guide the appropriate choice of transformation from a data-driven perspective. The best transformation metric has the least stress score. The Shepard diagrams allow us to visualise how well our MDS configuration fits our dissimilarity matrix.


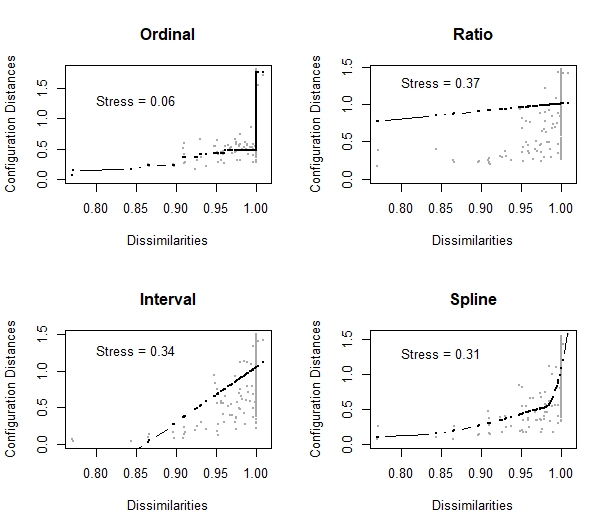


Figure S3: Shepard diagrams associated with the suboptimal population cohort.


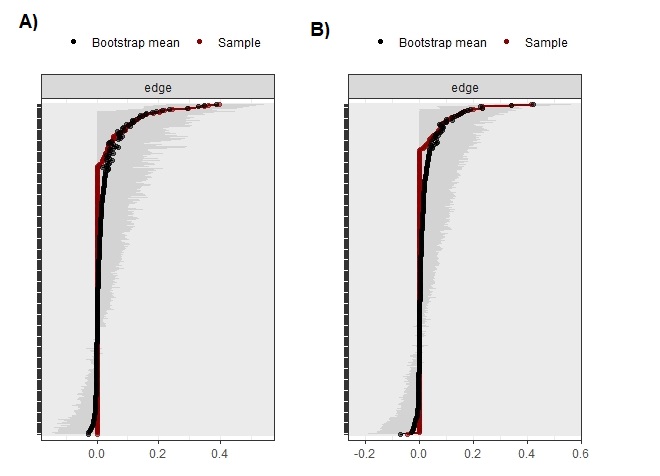


Figure S4: Bootstrapped confidence intervals of estimated edge-weights for the Graphical LASSO model estimated networks of the SHSQ-25 questionnaire for the optimal and suboptimal population cohorts. The red line indicates the sample values and the gray area the 95% bootstrapped confidence intervals. Each horizontal line represents one edge of the network, ordered from the edge with the highest edge-weight to the edge with the lowest edge-weight. In the case of ties, the mean of the bootstrap sample was used in ordering the edges. The y-axis labels have been removed to avoid cluttering (Epskamp, Borsboom, & Fried, 2012^[[1]](#footnote-1)^)


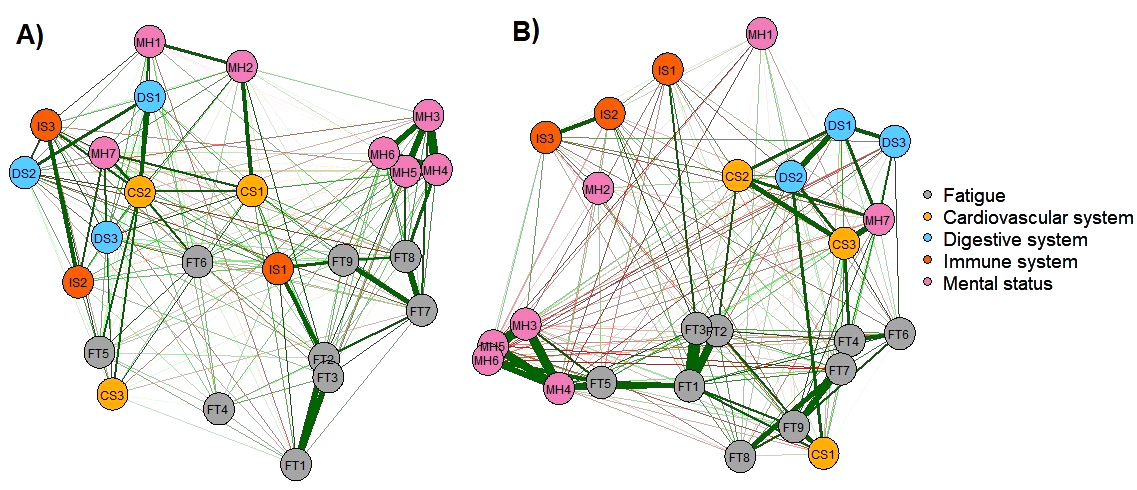


Figure S5: Full network structures of the relationships among the 5 health domains of SHSQ-25 for two classified population cohorts **A)** optimal status **B)** suboptimal status. Green and red edges indicate positive and negative associations respectively.

| **Optimal** | **Suboptimal** |
| --- | --- |
| 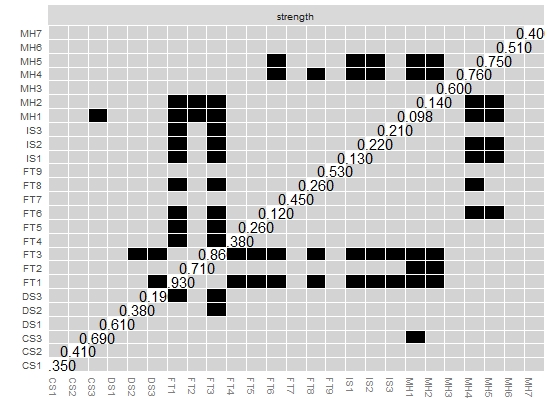 | 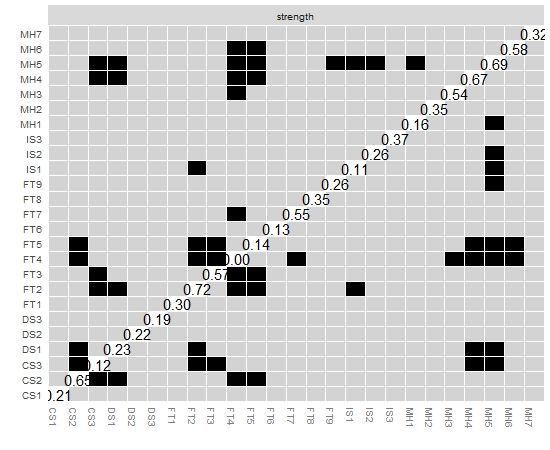 |

Figure S6: Bootstrapped difference tests (α = 0.05) between edge-weights in the estimated networks in Figure S4. Gray boxes indicate edges that do not differ significantly from one-another, and black boxes represent edges that do differ significantly from one-another. The numbers in the main diagonal represent the value of node’s strength.


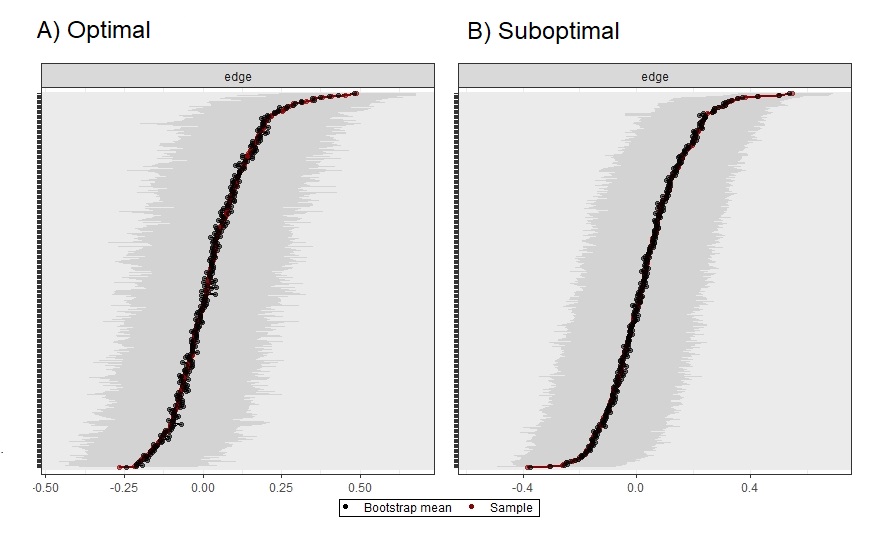


Figure S7: Bootstrapped confidence intervals of estimated edge-weights for the full estimated networks of the SHSQ-25 questionnaire for the optimal and suboptimal population cohorts in Figure S5. The red line indicates the sample values and the gray area the 95% bootstrapped confidence intervals. Each horizontal line represents one edge of the network, ordered from the edge with the highest edge-weight to the edge with the lowest edge-weight. In the case of ties, the mean of the bootstrap sample was used in ordering the edges. The y-axis labels have been removed to avoid cluttering

1. Epskamp, S., Cramer, A. O., Waldorp, L. J., Schmittmann, V. D., & Borsboom, D. (2012). Qgraph: Network visualizations of relationships in psychometric data. *Journal of Statistical Software*, 48(4), 1–18. [↑](#footnote-ref-1)
