# Supplementary material for: Conceptualised psycho-medical footprint for health status outcomes and the potential impacts for early detection and prevention of chronic diseases in the context of 3P medicine
Source: EPMA J. 2023 Nov 8;14(4):585–99. doi: 10.1007/s13167-023-00344-2 (PMC10713508; doi:10.1007/s13167-023-00344-2)
Supplement: Supplementary file 2 — Supplementary file2 (DOCX 18 KB) [file 13167_2023_344_MOESM2_ESM.docx]

***Working hypothesis in the framework of PPPM***

The suboptimal health status questionnaire-25 (SHSQ-25) is a unique medical psychometric diagnostic tool for the early detection of metabolic diseases. The development of the medical psychometric instrument SHSQ-25 characterises the paradigm shift from reactive medicine to a more proactive approach that personalises the prevention, stratification, diagnosis, and treatment for specific patients, a concept known as predictive, preventive, personalised medicine (PPPM). The early recognition of individuals who are at risk of developing diseases would promote tailored intervention to optimise their health outcomes.

The synaptic connections between the 25 symptomatic items and their relevance in supporting the early monitoring and prevention of chronic diseases within the framework of predictive, preventive, and personalized medicine (3PM) remain unclear. In the pursuit of 3PM, data generated from SHSQ-25 for the general population would be deemed as ‘big data’ and therefore there is the need for robust and tailored computational methodologies that can identify the patterns of interrelatedness that exist within the five subscales to fully understand the synaptic transmission between optimal and suboptimal health outcomes. For many years, conventional statistical methods have been used to establish the relationship between health domains in the SHSQ-25. However, data over-fitting, the curse of dimensionality and multicollinearity are only a few drawbacks that prevents effective interrogation of big data. Towards this objective, computational methodologies that support data visualisation would provide opportunity for quick, efficient, and real-time monitoring of the synaptic transmissions between the health status states. Thus, we premised the present study on the hypothesis that network analysis can provide a single time point photogenic image that highlights the patterns of interactions between health status outcomes.

To be able visualise and identify the relationships between several symptoms and their combinations associated with health status outcomes would create a comprehension for patients’ risk stratification and diagnostic paths of diseases. Network analyses have allowed researchers to visualise and identify the complex relationships between several symptoms and their combinations associated with diseases’ progression. In network analyses, symptoms are computationally analysed, rather than relying on global scores from scales. It provides data on the prognosis or outcomes for patients and utilises sum scores to demonstrate how symptoms are related to a condition. This allows for the detection of pathways by which symptoms influence each other, creating a profile of syndromes. This baseline study will inform the future development of symbiotic network visualisation tools based on time varying psychometric data from SHSQ-25 screenings. If we can construct distinctive SHSQ-25 footprint for SHS, then have demonstrated the feasibility of creating dynamic visualizers of the evolutionary trends in the relationships between the domains of SHSQ-25 and health status outcomes, which can become a new target for 3PM with potential benefits in the healthcare systems. Thus, this approach will bring the promise of 3PM closer to translational reality from SHSQ-25 screenings to detection and possible reversal of life-threatening conditions. More importantly, the SHS footprints would provide comprehensive evidence-based medicine that relies on sound theoretically conceptualized model that is convenient, quicker, and cheaper, and would achieve personalized prediction and monitoring of suboptimal health conditions, which are precursors for chronic diseases.

***Innovation towards the***

- 1. ***predictive approach,***

The SHSQ-25 has thus far been shown to identify the early signs of risk in a general population. This would be beneficial, as screening would target a specific population, while reducing screening for lower risk individuals. For example, given the genetic diversity within a population, patients with metabolic conditions may have the same clinical profile (e.g., body mass index, glycated haemoglobin, age, plasma lipid status) but will respond differently to a treatment. Thus, healthcare professionals must examine the genetic makeup of each individual and develop therapies that are specific to that individual. Moreover, people who complete the SHSQ-25 will know their risk, which in turn would empower them to modify their lives in a manner that would reduce their risk or protect them from transitioning into a disease. SHS screenings at healthcare facilities observed over time would provide rich data gathered economically to build robust predictive models to classify optimal and sub-optimal status, as demonstrated in this study. The proposed method of analysis provides opportunity to provide holistic care, which will be cost-effective in predicting individualised health status.

- 1. ***targeted prevention and***

From the lens of 3PM, the current status quo of treating chronic conditions after symptom onset is a delayed response. Substantial and compelling evidence have shown that the disability and complications linked to chronic conditions can be prevented or delayed. Nearly a third of people with chronic conditions are unaware they have it and the effectiveness of screening for such conditions remains unclear. The path to developing a chronic disease can be long, even up to 30 years, and the SHS screenings at healthcare facilities observed over time help to identify individuals with sub-optimal health status. The accurate prediction of individuals at risk would lead to the initiation of interventions before symptoms worsen.

- 1. ***personalisation of medical services.***

Holistic care must be viewed from the perspective of primary, secondary, tertiary, and quaternary care. The foundation of this concept is that the manifestation, severity, long term effects, respectively, of a particular chronic condition can be prevented. To combat the delayed intervention, untargeted medication, overdosed and poisoned patients, and poor therapy, primary care practitioners must be able to recognise and manage SHS. This must go beyond the usual recognition of physical deterioration, but also practitioners must pay attention to the psychological state of the individual. The SHSQ-25 offers the opportunity to recognise the reversible damage in an individual and serves as a catalyst to establish appropriate interventions for risk reduction.

***How does the presented innovation go beyond the state of the art contributing to the paradigm shift from reactive medicine to PPPM?***

We have demonstrated the discriminative capabilities of the SHSQ-25 to categorise individuals into optimal and suboptimal health status and have constructed single time point photogenic representations of their distinct relationship patterns in network analysis. Data visualisation in medical research has become useful in disease diagnosis, as it provides opportunity for quick, efficient, and often real-time monitoring of the aetiological processes of diseases. The ability to visualise and identify the relationships between several symptoms and their combinatory associations with diseases would create an in-depth comprehension for patients’ risk stratification and diagnostic pathways for diseases. The path to developing a chronic disease can be long, even up to 30 years, and the SHS screenings at healthcare facilities observed over time would be economical. Their analyses would have potential to substitute clinical biomarkers in metabolic diseases diagnoses, which are often expensive and time-consuming. Thus, relying on the outcomes of the SHS score can be a cost-effective way of determining the health status, and initiate an intervention before symptoms worsen.

Using network methodology, we have been able to discern which specific combinations and patterns of the 25-items in the SHSQ-25 are behind a subclinical health. We have demonstrated the feasibility of using network analysis based on time varying psychometric data to create dynamic visualizers of the temporal evolutionary trends in the relationships between the domains of SHSQ-25. This will provide in-depth comprehension of the conceptual model for suboptimal health status and its association with the aetiological processes of metabolic diseases. The findings are important because at-risk individuals can be prioritised for monitoring and targeted intervention can begin before their symptoms reach an irreversible stage.

This baseline study has extended the narrative and provided extensive investigation of the cross-sectional footprints of the 5 SHS dimensions among a suboptimal population cohort, providing many insights into the feasibility and opportunities of longitudinal study designs. We have demonstrated the feasibility of creating dynamic visualizers of the evolutionary trends in the relationships between the domains of SHSQ-25 relative to health status outcomes. This will provide in-depth comprehension of the conceptual model to inform personalised strategies to circumvent suboptimal health status.
